# Glutamic Acid Decarboxylase Antibody Unexpectedly Detected During Recovery Phase in Three Patients With Voltage-Gated Potassium Channel Antibody-Related Autoimmune Encephalitis

**DOI:** 10.7759/cureus.93345

**Published:** 2025-09-27

**Authors:** Aengela J Kim, William J Frem, Alekhya Bommireddipalli, Arin Boghoz, Fatin Aylia, Ifeanyichukwu Ozobu, Sara G Haroutunian, Rachel Williams, Jessica Alkana, Jiyang Lee, Antonio K Liu

**Affiliations:** 1 Internal Medicine, Adventist Health White Memorial, Los Angeles, USA; 2 Neurology, University of Texas Southwestern Medical Center, Dallas, USA; 3 Family Medicine, California Hospital Medical Center, Los Angeles, USA; 4 Medical Genetics, National Institutes of Health, Bethesda, USA; 5 Medicine, University of Central Florida, Orlando, USA; 6 Neurology, Adventist Health White Memorial, Los Angeles, USA; 7 Neurology, Loma Linda University School of Medicine, Loma Linda, USA

**Keywords:** anti-glutamic acid decarboxylase, autoimmune encephalitis and atypical presentations, contactin-associated protein-like 2, lgi1 antibody autoimmune encephalitis, morvan syndrome, voltage-gated potassium channel autoimmune encephalitis

## Abstract

Voltage-gated potassium channel (VGKC) and glutamic acid decarboxylase (GAD) antibodies are increasingly tested in patients with suspected autoimmune encephalitis (AE) and other neurological diseases, yet their clinical significance, especially when detected outside of classic contexts, remains poorly defined. This case series of three patients explores an unusual pattern of antibody dynamics in VGKC antibody-positive and leucine-rich glioma-inactivated 1 (LGI1) and contactin-associated protein-like 2 (CASPR2) antibodies-negative AE. All patients underwent immunotherapy based on clinical suspicion and improved, and had delayed emergence of GAD antibodies during recovery phases.

The key observations were that VGKC antibody levels were elevated during the acute presentation and either declined or normalized following treatment. GAD antibodies were absent during acute illness but emerged during the post-treatment/recovery phase, when patients were already recuperating. No patient developed syndromes classically associated with GAD antibodies (e.g., worsening encephalitis, stiff person syndrome, and refractory seizures). LGI1 and CASPR2 antibodies were consistently negative throughout all tested phases.

These findings bring up the questions of the role(s) GAD antibody may play in the disease processes in AE and other neurological diseases, including the possibility of GAD antibody positivity not necessarily indicating active or causative disease, and suggest it may, in some contexts, represent a phenomenon of immune remodeling or non-specific activation. Additionally, these cases reinforce the potential clinical utility of initiating immunotherapy based on phenotype and clinical suspicion, particularly in VGKC-positive, LGI1/CASPR2-negative presentations, while emphasizing the need for more nuanced interpretation of antibody dynamics over time.

## Introduction

Encephalitis encompasses a wide spectrum of inflammatory disorders of the central nervous system, traditionally attributed to infectious causes. However, over the past two decades, it has become increasingly evident that a substantial proportion of cases previously labeled “idiopathic” or “viral” are in fact driven by immune mechanisms rather than pathogens. This recognition has transformed both diagnostic and therapeutic approaches, highlighting a distinct category now referred to as autoimmune encephalitis (AE). AE is a noninfectious inflammation of the brain mediated by specific autoantibodies. In the past 15 years, there have been major developments in our understanding of AE, including the identification of an expanding list of specific antibodies and the increasing availability of corresponding commercial tests. The clinical spectrum is broad, ranging from altered mental status, medication-refractory seizures, and movement disorders to cognitive decline, autonomic dysfunction, and psychiatric symptoms. Multiple triggers have been identified, including post-infectious and paraneoplastic processes; however, many cases remain idiopathic or cryptogenic. Proposed mechanisms include molecular mimicry, direct autoantibody production in tumors, infection or inflammation-induced bystander immune activation, and certain genetic predispositions [1].

AE is increasingly recognized as a significant cause of encephalitis, with some studies suggesting its prevalence may be comparable to infectious etiologies, such as herpes simplex virus (HSV) encephalitis. However, many clinically suspected cases of AE do not meet all established diagnostic criteria, potentially leading to underestimation of its true prevalence [1]. A variety of neuronal antibodies have been linked to AE and related neurological syndromes. Two of the studied antibodies are those against glutamic acid decarboxylase (GAD) and the voltage-gated potassium channel (VGKC) complex. These antibodies are associated with distinct clinical and pathological features and may offer insights into underlying mechanisms and treatment responses.

GAD is an intracellular enzyme primarily expressed in neurons and pancreatic beta cells, where it converts the excitatory neurotransmitter glutamate into the inhibitory neurotransmitter gamma-aminobutyric acid (GABA), thus modulating neuronal excitability [2]. Autoantibodies targeting GAD have been implicated in a spectrum of neurological disorders, including stiff person syndrome (SPS), cerebellar ataxia, limbic encephalitis, and medication-refractory status epilepticus [3,4]. The first clinical association of elevated GAD antibodies was described in 1988, and since then, GAD antibody-spectrum disorders (GADSD) have been recognized, encompassing autoimmune neurological diseases marked by excessive neuronal activity [5]. Despite this, several critical gaps remain in our understanding of GAD antibodies. There is limited data on (1) the presence of GAD antibodies in asymptomatic individuals, (2) the sensitivity and specificity of GAD antibody assays in various contexts, (3) potential false positives, and (4) the dynamics of antibody levels over the disease course or recovery phase. Of note, GAD antibody is also associated with diabetes mellitus, but that is beyond the scope of this article.

VGKCs are membrane proteins critical for maintaining neuronal membrane potentials. The immune response targeting VGKC can extend beyond initial epitopes via epitope spreading, disrupting normal neural excitability [6,7]. VGKC antibodies have been associated with limbic encephalitis [8] and Morvan syndrome [9]. More refined research has revealed that pathogenic antibodies often target specific components of the VGKC complex, notably leucine-rich glioma-inactivated 1 (LGI1) and contactin-associated protein-like 2 (CASPR2), and each is associated with distinct clinical features and variable treatment responses [10]. While LGI1 and CASPR2 antibodies are more specific markers within the VGKC complex, a subset of patients presents with VGKC antibody positivity without positivity for these subtypes, complicating diagnosis and management. Many experts view VGKC antibody positivity without LGI1 or CASPR2 as nonspecific and even advocate ceasing VGKC testing in isolation [11]. Furthermore, concerns about overdiagnosis have prompted calls for stricter diagnostic criteria to avoid mislabeling patients based solely on VGKC positivity [12]. Nonetheless, VGKC antibody-positive cases without LGI1 or CASPR2 seropositivity still occur, and there is no consensus or guideline on their management when no other etiology is found [13].

Overall, the roles and pathological mechanisms of GAD and VGKC antibodies in AE remain incompletely understood. Although some patients have been reported with both antibody types simultaneously [14,15], the clinical significance and underlying reasons for this overlap have not been thoroughly investigated. Most literature focuses on the distinct clinical features of either GAD or VGKC antibody-associated syndromes rather than their coexistence [16]. To our knowledge, no previous studies have documented cases where GAD antibodies emerge only during the recovery phase following an acute VGKC antibody-related illness. In this paper, we present three such cases to highlight this unusual temporal antibody pattern and explore its clinical implications.

## Case presentation

Case 1

**Figure 1:**
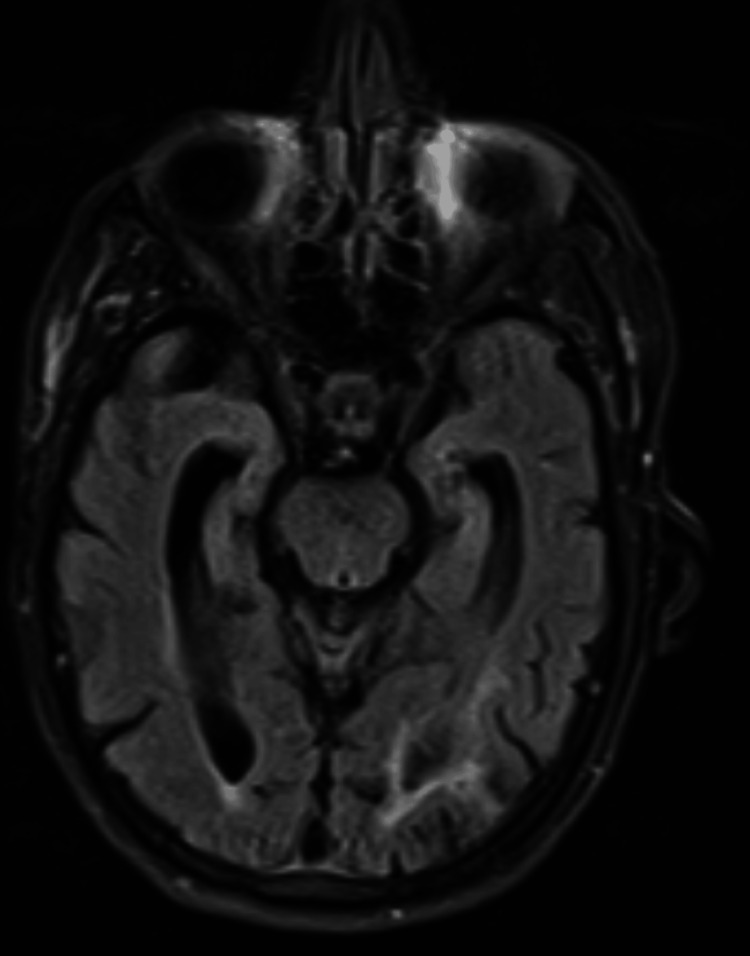
Negative fluid-attenuated inversion recovery (FLAIR) sequence of brain MRI at the level of the hippocampus.

A 77-year-old male presented to our emergency department (ED) for worsening altered mental status over one month. Before this, he was independent at baseline. He had no known medical history. He had no diabetes mellitus (DM). He had become increasingly incoherent and had been found wandering the streets. He could no longer carry on a normal conversation and had been having decreased oral intake. His condition worsened, and he had become bedbound prior to admission. In the ED, he was a thin and cachectic man. His vital signs were normal. His temperature was 37°C, blood pressure was 158/99 mmHg, and heart rate was 89. He was not oriented. He made noise with constant moaning and groaning. He readily withdrew all four extremities to noxious stimuli applied to nail beds in a non-focal manner. Startling did not trigger any worsening and did not cause any stiffness. His white count, chemistry, hematologic studies, renal function, and liver functions were all unremarkable on laboratory studies. His head CT and brain MRI were negative for an acute event and inflammation (Figure 1). Cerebrospinal fluid (CSF) analysis showed 1 white blood cell (wbc) per mm^3^ and protein of 31 mg/dL. CSF cultures and viral and fungal studies were all negative. Serum autoimmune encephalitis antibody panel test 3006051, run by Associated Regional and University Pathologists, Inc. (ARUP), was sent on day two of hospitalization. Since this test had a turnaround time of three weeks (at the time of admission, it is faster nowadays), we initiated treatment shortly after admission due to a high degree of suspicion for autoimmune encephalitis. He received a course of methylprednisolone 1000 mg intravenous (IV) daily for five days and intravenous immunoglobulin (IVIG) at 400 mg/kg/day for five days. Eventually, the test came back with one single antibody positive; VGKC antibody was found to be 142 pmol/L (reference: 0-31), upon which, a dose of rituximab 375 mg/m^2^ was also administered. The patient slowly improved with treatment. By hospital day 40, he was oriented to name and place, he could follow commands, and was able to ambulate with a walker and minimal assistance from physical therapy. A repeat antibody testing (sent 61 days after the first test) came back with a lowered VGKC antibody level at 71 pmol/L (reference: 0-31), but GAD antibody (which was normal the first time) was measured at 37.6 IU/ml (reference <5.0) (Table 1). The patient was eventually transferred to a skilled nursing facility. During the course of this hospitalization, the patient never demonstrated any sign or symptom of stiff person syndrome, and a basic malignancy workup was negative. On a follow-up phone call, six months post discharge, we found out that the patient had returned home, generally independent of activities of daily living, with minimal supervision. Eighteen months later, an ED visit for unrelated issues found the patient being alert and oriented (x2), with non-focal examination; however, ambulatory and independent. ILI-2 and CASPR-2 antibodies were all less than 1:10 in all testing.

**Table 1:** Patient data and laboratory values. CSF: cerebrospinal fluid; VGKC: voltage-gated potassium channel; GAD: glutamic acid decarboxylase; HSV: herpes simplex virus.

Item/patient	Patient 1	Patient 2	Patient 3	Reference
CSF wbc	1	10 wbc/mm^3^	3 wbc/mm^3^	<5 wbc/mm^3^
CSF protein	31	23 mg/dL	44 mg/dL	14-40 mg/dL
VGKC 1^st ^	142 pmol/L	58 pmol/L	42 pmol/L	Negative <31, intermediate = 32-87, positive > 88 pmol/L
VGKC 2^nd^	71 pmol/L	86 pmol/L	91 pmol/L	Same as above
VGKC 3^rd^	NA	34	NA	Same as above
GAD 1^st^	<5 IU/mL	<5 IU/mL	<5 IU/mL	<5 IU/mL
GAD 2^nd^	37.6 IU/mL	70.7 IU/mL	134.5 IU/mL	Same as above
GAD ^3rd^	NA	<5 IU/mL	NA	Same as above
Days between 1^st^ and 2^nd^ tests	61 days	200 days	367 days	NA
Days between 2^nd^ and 3^rd^ tests	NA	206 days	NA	NA
Other positive	NA	Low amphiphysin 1^st^ test	CSF HSV IgG, both tests	NA
MRI brain	Negative	Negative	Negative	NA

Case 2

**Figure 2:**
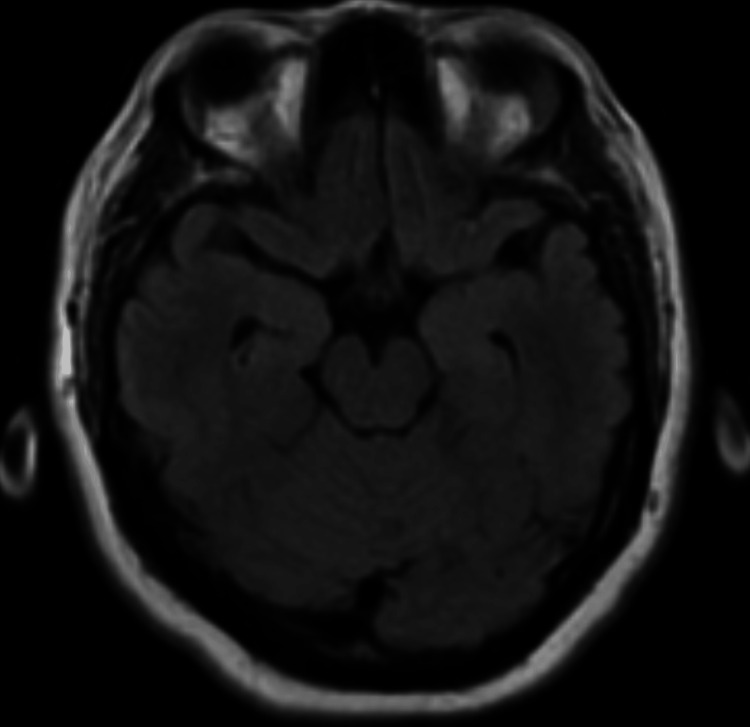
Negative fluid-attenuated inversion recovery (FLAIR) sequence of brain MRI at the level of the hippocampus in mesial temporal lobes.

A 43-year-old female with obesity and type 2 DM presented to the ED with the sudden onset of right facial muscle twitching and involuntary right eyelid spasm. Her vital signs were stable, temperature was 36.5°C, blood pressure was 150/87, and heart rate was 85. There was no altered mental status initially. She had a right blepharospasm and right hemifacial spasms, and myokymia. She had intact extraocular movements, and the neurological examination below the neck was non-focal and unremarkable. CT and MRI of the brain were negative (Figure 2). CSF analysis revealed 10 wbc/mm^3^ and CSF protein at 23 mg/dL. She had also been having ongoing insomnia for three weeks. She began to experience hallucinations and disorientation a few days after admission. CSF analysis yielded 10 wbc/mm^3^, lymphocytic predominant, CSF protein of 23 mg/dL. With a working diagnosis of Morvan syndrome, the patient was symptomatically managed with oral clonazepam, a five-day course of methylprednisolone 1000 mg IV, and IVIG 400 mg/kg/day for five days. A serum autoimmune encephalitis antibody panel from ARUP revealed VGKC of 58 pmol/dL and a low amphiphysin level (no titer available). Her symptoms improved two weeks after admission. Repeated intentional startling did not trigger any symptoms, and she was discharged home after a one-month stay. Her facial spasms were clonazepam responsive but still visible. She presented to the ED roughly six months later with a flare-up of facial spasms. She reported interval improvement but no resolution prior to worsening. Her examination showed normal mentation but involuntary facial spasms and blepharospasms, which were worse than at the time of her previous discharge. Repeat labs were obtained, and this time, VGKC antibody was 86 pmol/L, while no amphiphysin was detected. GAD antibody came back positive at 70.7 IU/mL (Table 1). She received another five-day course of methylprednisolone 1000 mg IV daily, five days of IVIG 400 mg/kg/day, and one course of rituximab 375 mg/m^2^. Her symptoms improved over the coming weeks, and she remained clonazepam responsive. Baclofen was added to further her symptomatic control. She was discharged home after three weeks of stay. She presented to the ED again with a minor flare-up six months later. Facial spasms were visible but were less than during her prior episodes. Her chief complaint was more focused on facial and body pain, and prior to knowing her VGKC and GAD levels, a course of five days of methylprednisolone 1000 mg IV and IVIG 400 mg/kg/day for five days was administered. Her symptoms, especially the pain, improved. She was discharged prior to receiving results of the serum autoimmune antibody panel, which showed VGKC of only 34 pmol/L, GAD less than 5 IU/mL, and no detectable amphiphysin. ILI-2 and LASPR2 were all less than 1:10 in all testing. A follow-up phone call three months later found the patient with minor symptoms, but functional, and with no indication to return to the ED.

Case 3

**Figure 3:**
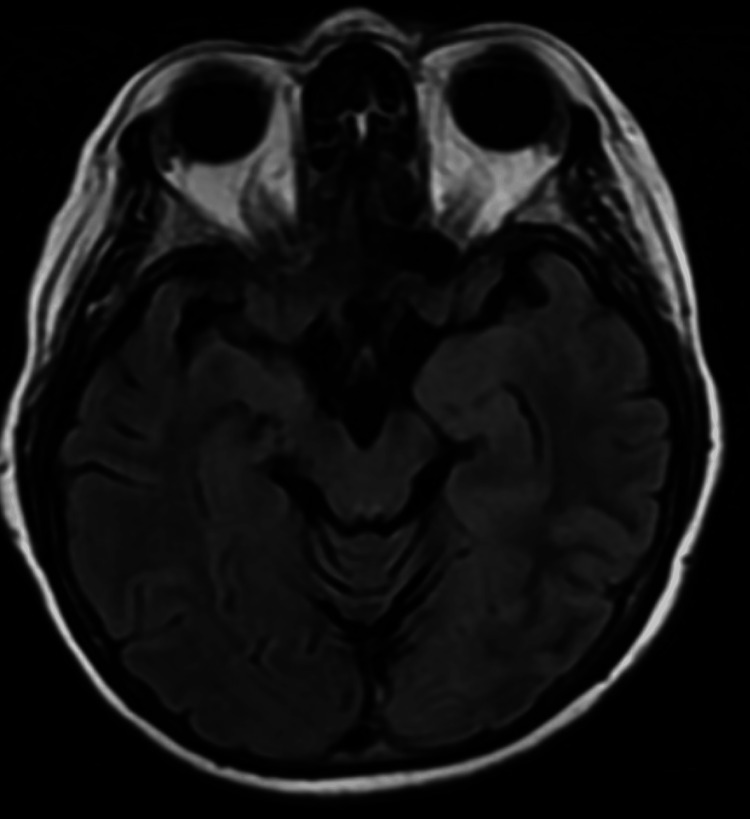
Negative fluid-attenuated inversion recovery (FLAIR) sequence of brain MRI at the level of the hippocampus in mesial temporal lobes.

A 67-year-old female with type 2 DM and hypertension came to the ED after two weeks of headaches, fevers, and altered mental status. Two prior ED visits elsewhere yielded no diagnosis, and therefore, treatment had not been initiated. She had a temperature of 100°F. While her other vital signs were stable, her blood pressure was 156/89 mmHg, and her heart rate was 84. She was only oriented to name and was experiencing active hallucinations. Cranial nerves, motor, sensory, and cerebellar examination were all non-revealing. CT and MRI of the brain were negative (Figure 3). CSF analysis revealed 3 wbc/mm^3^ and protein of 44 mg/dL. CSF herpes simplex virus (HSV) 1/2 antibody IgG was 1.38 IV. Despite IgM being negative, a course of IV acyclovir was administered. The autoimmune encephalitis panel was sent to ARUP and came back positive for VGKC at 42 pmol/L. A five-day course of methylprednisolone 1000 mg daily, a five-day course of 400 mg/kg/day of IVIG, and a single dose of 375 mg/m^2^ rituximab were administered. Her mental status greatly improved over the next few weeks, and she was eventually discharged home. She presented to the ED one year later with acute encephalopathy likely secondary to a urinary tract infection (UTI). Her family reported she had returned to her usual baseline three months after her prior discharge. We repeated both a CSF analysis and the same autoimmune antibody panel from ARUP. CSF WBC was 0, CSF protein was 47 mg/dL, and CSF HSV 1/2 IgG antibody was 1.63 IV. Her brain MRI was again negative. Her mentation rapidly returned to baseline with antibiotics for her UTI, and she was discharged after three days of hospitalization; no immunomodulation therapy was indicated or administered. Lab results returned weeks after her discharge. This time, 367 days after the original test, VGKC was 91 pmol/L and GAD was perplexingly positive at 134.5 IU/mL. ILI-2 and CASPR2 antibodies were all less than 1:10 in all testing. A telephone call follow-up found the patient back at her baseline.

## Discussion

Our central observation, i.e., the unexpected appearance of GAD antibodies during the recovery phase in patients with VGKC-positive AE, necessitates a deeper exploration of the typical patterns associated with these antibodies. When antibodies emerge in unusual phases of illness, as seen with GAD in our patients, interpreting their clinical relevance requires more than serological and clinical correlation. As our cases suggest, not all antibody detections should be equated with active autoimmune pathology, particularly in the absence of correlating symptoms. In our cases, all patients showed VGKC antibody positivity, while LGI1 and CASPR2 antibodies were negative. An extensive workup was done, and there was no other autoimmune disease or alternative explanation identified in these three cases. All three patients improved with immune modulation treatment, so the delayed emergence of GAD antibodies while they were recovering generated three major questions. First, what is GAD’s role in neurological diseases? Secondly, can GAD antibody positivity during recovery represent a secondary, unrelated process, or does it suggest a broader post-autoimmune or post-infectious autoimmune shift? Lastly, what is the actual role of VGKC antibodies in disease in general?

GAD- vs. VGKC-antibody-related AE

Different autoantibodies in AE are associated with distinct clinical features, prognoses, and responses to treatment, making their identification clinically relevant. A study by Malter et al. involving 53 limbic encephalitis patients showed GAD-positive patients were younger and typically presented with isolated seizures, while VGKC-positive cases displayed broader limbic features. Importantly, after corticosteroid treatment, GAD antibody levels remained persistently high in all cases, while VGKC antibodies normalized in most. None of the GAD antibody group became seizure-free, unlike the VGKC patients [17].

In VGKC antibody-associated AE, studies show significant early impairments in memory, executive function, and processing speed. However, these domains tend to recover well following immunotherapy, with many patients regaining normal processing and executive functions, though some memory deficits may persist [10,17]. GAD antibody-positive AE, in contrast, usually demonstrates a more chronic and refractory trajectory. Patients often show less impairment in learning and memory early on, and they also exhibit only a modest improvement with immunotherapy [16-18]. GAD antibody positivity is linked to persistent antibody presence, slower recovery, and structural changes suggestive of chronic inflammation and neurodegeneration [17]. In general, VGKC antibodies often appear and resolve in tandem with clinical symptoms, whereas GAD antibodies persist, hinting at a causative role in ongoing inflammation and tissue damage [19].

The uncertain role of GAD antibodies in neurological disease

The timing of GAD antibody detection in our cases, emerging only during the recovery phase, raises important questions about their clinical significance. This temporal pattern suggests a few different possibilities, including that neurological disease with GAD involvement may be that of a longer symptomatic process, that GAD antibodies may not reflect the active or initial disease process but rather represent a phenomenon occurring after the acute neurological syndrome has already begun to resolve, or even that GAD antibodies may be an innocent bystander.

The latter perspective is supported by a study of neurocritical care patients in which patients responded well to immunotherapy, but GAD antibody titers did not decline in parallel with clinical improvement, suggesting a possible lack of pathogenic correlation [19]. Conversely, there are case series in which GAD antibody titers decreased following immunotherapy alongside symptom improvement, suggesting that the dynamics may vary depending on the context or disease subtype [20].

One particularly illustrative case described a patient with an incidental GAD65 antibody finding, with no neurological symptoms initially. Eighteen years later, the patient developed autoimmune limbic encephalitis and refractory temporal lobe epilepsy. At that time, both GAD65 and GAD67 were present in serum and CSF [21]. These data collectively highlight the temporal complexity of GAD antibody expression. These may suggest that GAD antibodies may reflect secondary immune activity, such as bystander activation, immune remodeling, or delayed immune memory responses, rather than active contributors to neuronal injury. This further supports the importance of interpreting GAD antibody findings within a broader clinical and temporal context, particularly when other hallmark features (e.g., seizures, SPS, and intrathecal synthesis) are absent.

A growing body of evidence highlights the complex and often ambiguous role of GAD antibodies in neurological conditions. While GAD antibodies are central to the diagnosis of certain syndromes like SPS, their presence outside of these well-established contexts is not always clinically meaningful. For example, in SPS, intrathecal GAD antibody production confirms a pathogenic role. In other conditions, such as epilepsy or cerebellar ataxia, there is no consensus on causality. Without this, a GAD antibody finding in serum alone should be interpreted cautiously, as it may be incidental or epiphenomenal [22].

Rare dual positivity: VGKC and GAD antibodies

Dual positivity for VGKC and GAD antibodies has been rarely reported in the literature. One case described a patient with cerebral palsy who experienced recurrent insomnia, visual hallucinations, and paranoia, and was found to be positive for both VGKC and GAD antibodies. The patient responded completely to IVIG treatment. However, the report did not document the timing or sequence of antibody positivity, limiting its relevance to understanding causality or temporal dynamics [23].

Our cases highlight a unique and under-reported pattern in which GAD antibodies emerge only during the recovery phase, following a VGKC-positive AE. This delayed GAD detection, in the absence of relapse or new neurological symptoms, adds to a growing body of evidence suggesting that GAD antibodies may not always be pathogenic, and perhaps sometimes reflect secondary or incidental immune phenomena rather than direct drivers of disease.

VGKC without LGI1 and CASPR2

In all three of our cases, LGI1 and CASPR2 antibodies were consistently negative on initial and repeat testing. This raises an important diagnostic challenge, as many experts advise caution in diagnosing AE based solely on VGKC positivity. In fact, VGKC antibodies, without LGI1 or CASPR2, are now widely regarded as non-specific, and some experts have even questioned whether VGKC testing should be used at all [11]. As an extensive workup has ruled out other alternatives in our three cases, VGKC positivity was beyond incidental.

A recently reported case of limbic encephalitis further illustrates the clinical significance of VGKC antibody positivity in the absence of LGI1 and CASPR2 antibodies. In this patient, VGKC antibodies were detected despite negative LGI1, CASPR2, and GAD-antibody testing. The patient experienced full symptom resolution following IVIG treatment, with one recurrence after 1.5 years that again responded well to IVIG [23]. This case adds to our cases, in that VGKC antibody positivity alone, even without the more specific LGI1 or CASPR2 subtypes, can be associated with a treatable AE and aligns with our observations of VGKC-positive, LGI1/CASPR2-negative patients.

Limitations and additional considerations

Our medical centers have experienced a surge in autoimmune neurological diseases in recent years [15]. A common feature among these patients is that they underwent multiple general medical and neurological workups without a clear diagnosis. The worsening of their symptoms eventually prompted testing for autoimmune antibodies. Consequently, the use of autoimmune neurological disease panels has increased, including repeat and follow-up testing. In our current report, the detection of these antibodies is undoubtedly influenced by this increase in testing, an example of detection bias.

The timeline of antibody testing in our report has limitations. These were not prospective studies, and symptom onset was often unclear or retrospective, making it difficult to determine the optimal timing of antibody appearance. Due to resource limitations and patient access issues, we were unable to perform third-round testing in cases 1 and 3.

The second case’s first VGKC level was only 58 pmol/L, which is intermediately positive, and even the second testing level reached only 86 pmol/L, just shy of the formal positive cutoff. Lacking any other alternative diagnosis, Morvan syndrome secondary to VGKC antibodies remained the working diagnosis. A potential confounding factor is the presence of low-level amphiphysin antibodies initially detected, but literature searches show no reports linking amphiphysin with symptoms such as myokymia or facial spasms. This is the only case in which we monitored VGKC and GAD antibodies on three occasions: VGKC was detected during the acute disease phase, then increased and eventually decreased back to normal. While the patient’s symptoms improved and were controlled with medication, a full return to baseline did not occur. Notably, the resolution of antibody levels did not correlate with symptom resolution, suggesting some residual symptoms may be permanent.

There are also additional considerations. Case 3 tested positive for HSV IgG in CSF. Previous exposure is the only conclusion that can be drawn. This raises another question: could prior exposure to HSV lead to the development of VGKC-related AE? HSV is better known for its relationship with N-methyl-D-aspartate (NMDA) encephalitis [24]. Less frequently, HSV has been associated with encephalitis, considered postinfectious with CASPR2 antibody, part of the VGKC complex [25].

This report does not seek to establish the pathogenic mechanisms of VGKC or GAD antibodies in encephalitis. Rather, it highlights a novel and under-reported pattern: the emergence of GAD antibodies during recovery, after improvement from an unrelated acute neurological syndrome. While a robust body of literature links GAD to autoimmune neurological syndromes, these findings do not apply here, as none of our patients had new altered mental status, seizures, stiff person syndrome, or relapse when GAD became detectable.

## Conclusions

Our unique observation of GAD antibody emergence during the recovery phase, without concurrent clinical relapse or typical GAD-associated symptoms, raises important questions about the pathogenic role of GAD antibodies. The findings challenge the conventional assumption that GAD positivity necessarily reflects active or causative autoimmune neurological disease. It presents the possibility that GAD antibodies may sometimes represent an epiphenomenon or delayed immunological marker unrelated to acute symptomatology.

AE is a potentially reversible condition that is underdiagnosed and demands timely and judicious use of costly and often non-benign immunosuppressive therapies. The consistent absence of LGI1 and CASPR2 antibodies in these cases, with positive VGKC antibodies, underscores the diagnostic uncertainty surrounding VGKC without its more specific subunits. While exclusion criteria for VGKC-related AE have been suggested to reduce misdiagnosis, clear inclusion criteria and treatment guidelines are needed for patients presenting with VGKC positivity alone. Until then, clinicians must continue to navigate these complexities with careful clinical judgment, as early treatment decisions are often guided by clinical suspicion rather than definitive biomarkers.
